# Prevalence and Risk Factors Associated with Intestinal Parasitic Infection among Primary School Children in Dera District, Northwest Ethiopia

**DOI:** 10.1155/2021/5517564

**Published:** 2021-09-21

**Authors:** Dires Tegen, Destaw Damtie

**Affiliations:** ^1^Dera Woreda Education Office, South Gondar Zone, Gondar, Ethiopia; ^2^Bahir Dar University, Department of Biology, College of Sciences, Bahir Dar, Ethiopia

## Abstract

**Background:**

Globally, over 600 million school children are living with intestinal parasites. The prevalence of intestinal parasitic infections (IPIs) among school children in Ethiopia and the Amhara region is 52% and 51%, respectively. The present study aimed to determine the prevalence and associated risk factors of IPIs among primary school children in Dera district, Northwest Ethiopia.

**Methods:**

A school-based cross-sectional study was conducted from December 2019 to February 2020. The study used a structured pretested questionnaire and stool tests to obtain epidemiological and disease data. Data were analyzed using appropriate univariate and multivariable logistic regression methods by statistical package for social science (SPSS) version 25.0.

**Results:**

Of the 382 students who were examined for IPIs, 238 (62.3%) (61.8% males, 62.8% females) were positive for one or more IPIs. One hundred thirty-six (35.6%), 98 (25.7%), and 4 (1.05%) were single, double, and triple infections, respectively. Out of the nine species of IPIs detected, *Entamoeba* sp. was the predominant species (29.6%) followed by hookworm (21.7%), *Schistosoma mansoni* (11.3%), *Taenia* sp. (9.2%), *Giardia lamblia* (5.2%), and *Ascaris lumbricoides, Hymenolepis nana,* and *Enterobius vermicularis* (4.2%) each, and *Trichuris trichiura* (0.5%). Family size greater than five (AOR = 1.8; 95% CI: 1.004, 3.13), open field school waste disposal (AOR = 15.88; 95% CI: 1.91, 132.1), and lack of knowledge about intestinal parasitic infection (AOR = 1.8; 95% CI: 1.1, 3.2) were the independent risk factors associated with the overall prevalence of IPIs.

**Conclusions:**

The prevalence of intestinal parasitic infection was high in the Dera district. Health education, extending school-based deworming, and mass treatments are recommended.

## 1. Introduction

Intestinal parasites are found in the gastrointestinal tracts of humans and other animals [1]. Globally, about 3.5 billion people are affected by parasitic infections. The annual morbidities and mortalities due to IPIs are estimated to be over 450 million and 200,000, respectively [2]. Morbidity and indirect effects of IPIs have a substantial impact on health and quality of life [3]. The World Health Organization (WHO) estimated that over 270 million preschool children and over 600 million school children are living in areas where the parasites are widely transmitted [3].

*A. lumbricoides, T. trichiura,* hookworms*, H. nana, S. mansoni*, *E. histolytica/dispar*, and *G. lamblia* are the major common IPIs reported globally [1, 3, 4]. The global trends of IPIs are 1.5 billion people with worms [5], 48 million people with amoeba, and 2.8 million people with giardia. The poorest and most deprived communities are highly affected [3].

In Ethiopia, 81 million people live in areas vulnerable to intestinal parasites, among which 25.3 million are school children (SC) [6]. Similarly, Ethiopia is a country with the lowest quality of drinking water supply (34%) and latrine coverage (7.1%) in the world [7]. Of the 7.1% latrine coverage, the latrine utilization level in Ethiopia is 50.02% [8]. Consequently, intestinal parasites and other communicable diseases are widely distributed in various localities [7]. As a result, intestinal parasites are common in most parts of Ethiopia, particularly in school children, and can reach up to 84.3% in some regions [9]. Studies in Ethiopia show the different prevalence of IPIs. For instance, the highest overall prevalence of IPIs is 84.3% in Debre Elias Primary Schools (North West Ethiopia) [9], and the lowest prevalence is from Babile town (13.8%) (Eastern Ethiopia) [10].

IPIs are regarded as serious public health problems as they can cause iron deficiency anemia, growth retardation in children, and other physical and mental health conditions [11]. Furthermore, they cause abdominal pain, cholangitis, obstructive jaundice, acute pancreatitis, hepatic abscess, central nervous system disorders, ocular disorders, and epilepsy [12, 13], and death in more extreme cases [14]. Infections with intestinal parasites cause gastrointestinal problems such as diarrhea, dysentery, vomiting, and lack of appetite [15]. Diarrhea, including that of parasitic origin, is among the most common illnesses which cause infant and childhood mortality in developing countries [16].

Age is one of the risk factors for infection by intestinal parasites. School children are at a high risk of intestinal parasite infection. Poor hygiene, low immune status, overcrowding, close contact with soil and with each other, lack of latrine, and inadequate provision of safe water in schools are associated risk factors for IPIs [17–19]. Poverty, poor environmental hygiene, and impoverished health services are some of the other factors that put school children at high risk for IPIs [11].

According to clinical reports from a health center in Dera district (Northwest Ethiopia), intestinal infections are among the top reasons why people visit health facilities (Dera Woreda Health Office Annual Report, 2019). Among these, children are the dominant group infected by IPIs. However, there was no previous study conducted on the prevalence and associated risk factors of IPIs among school children in the current study area. Therefore, the present study aimed to assess the prevalence of IPIs and their risk factors among school children in the primary schools of Dera district, Northwest Ethiopia.

## 2. Materials and Methods

### 2.1. Study Area

**Figure 1:**
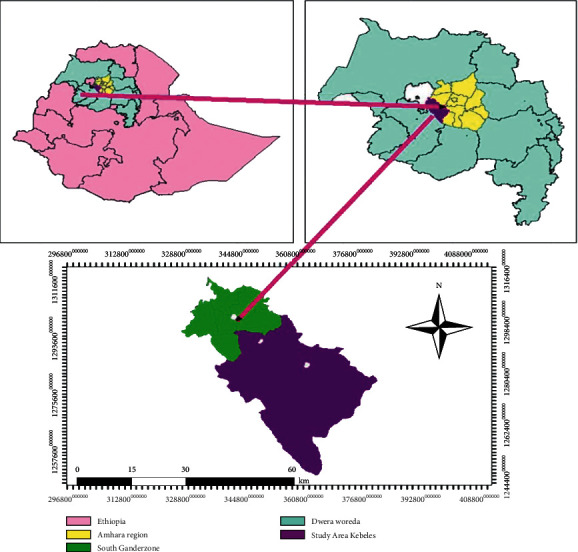
Location map of the study area.

This study was carried out in five primary schools from Dera district, South Gondar Zone, Amhara region, Northwest Ethiopia (Figure 1). It is located 535 km northwest of Addis Ababa (the capital city of Ethiopia) and 42 km away from Bahir Dar. Its geographical location is 11° 43′ 0″ N and 37° 38′ 0″ E and its elevation ranges from 1,560 to 2,600 m above sea level. Its mean annual temperature and rainfall are 26°C and 1250 mm, respectively. Agroecologically, 85% of the district is lowland and the remaining 15% highland. Based on the 2007 projected national housing and population census, the total population of the district was 248,464 (126,961 males, 121,503 females). The majority of the population (93.25%) lived in rural and 6.75% in urban areas. The main economic activity of the people in this area was agriculture [20].

There are 11 health centers and 36 health posts in the district. Health posts are assigned to implement the health extension program. Health Extension Workers (HEWs) spend 50% of their time visiting families in their homes and performing outreach activities in the community. HEWs are well-trained to provide first aid; treat diseases like malaria, dysentery, IPIs, and other ailments; and refer complicated cases to the nearest health center [21]. The district is rich with surface and groundwater resources such as Lake Tana and Abay River. However, access to clean water is limited. Thus, the community depends on various unprotected water sources.

### 2.2. Study Design

A school-based cross-sectional study was conducted from December 2019 to February 2020 to determine the prevalence of IPIs and associated risk factors among primary school students of Dera district, Northwest Ethiopia.

### 2.3. Study Population

The study population was all students enrolled in the five selected primary schools (from grades 1 to 8) 2019/2020 academic year. The number of students in each school was as follows: Gibtsawit (761), Enbosa Maseria (685), Wagira (752), Mirafe Mariam (1318), and Korata (1277), which gives the total number of 4793 (2455 males, 2338 females).

### 2.4. Sample Size Determination

The sample size was determined using the single population formula *n* = (*Z*^2^*p*(1 − *p*)/*d*^2^) [22]. The estimation of the sample size was based on population and previous studies on the prevalence of IPIs in the study area, where *n* = sample size; *Z* = 1.96 at 95% confidence level; *P* = estimated prevalence rate 50% = 0.5, and *d* = margin of error 0.05. Since there was no similar study previously conducted in the area, a 50% prevalence rate of IPIs was taken assuming that IPIs are significantly prevalent among students in Gibtsawit, Mirafe Mariam, Wagira, Korata, and Enbosa Maseria primary schools. Accordingly, the total sample size was

(1) $$\begin{array}{c} n=\frac{1.96^{2}\;0.50\left(1-0.50\right)}{0.05^{2}}=384\text{ students}. \end{array}$$

The researchers proportionally selected 384 study participants from the five schools. The sample distribution per each school was Gibtsawit (61), Korata (102), Wagira (60), Mirafe Mariam (106), and Enbosa Maseria (55).

### 2.5. Sampling Methods

The five target schools were randomly selected from the 114 schools in the Dera district and the 384 students were selected using a simple random sampling method. The alphabetically arranged list of all students from the five schools (grades 1–8) served as a sampling frame. The respondents were grouped into three age categories 6 to 11 years (middle childhood), 12 to 18 years (early adolescence), and 19 to 21 years (late adolescence) [23].

### 2.6. Questionnaire Survey

Trained data collectors collected the information (sociodemographic characteristics, child behavioral characteristics, and past medical history) of every participant using interviews of structured questionnaires first developed in Amharic language and translated back to English while encoding the data. Data collectors also made clarifications of ambiguous questionnaires to children. The questionnaires were pretested and validated. The data collectors also checked the fingernail cleanness and the shoe-wearing habit of each student. The criteria for clean hands were properly trimmed fingernails, physically clean hands during observation, and handwashing habits after touching any dirt material and before eating. The data collectors considered the students knowledgeable about IPIs if they correctly mentioned at least two IPIs with their etiologic agents, modes of transmission, and prevention methods.

### 2.7. Stool Sample Collection

Formed and semi-formed fresh stool samples were used for parasitological analysis. The children were trained on how to collect the stool samples hygienically and advised to bring their own 3 to 5 g fresh stools in labeled collection cups along with applicator sticks. Parents were engaged for children aged 6–11.

### 2.8. Stool Sample Examination

Stool samples were examined under wet-mount and formol-ether concentration techniques. A portion of each of the stool samples was processed and examined microscopically using direct wet-mount and formol-ether concentration techniques based on the procedures in WHO guidelines [24]. All developmental stages of the parasites (cyst, egg, larvae, and adult) were recorded.

### 2.9. Direct Microscopy (Wet Mount)

A direct wet-mount technique was processed by conventional iodine. About 2 g of each stool sample was emulsified with 3–4 ml normal saline (0.85% NaCl solution). Then a drop of the emulsified sample was placed on a clean microscopic glass slide to observe the presence of trophozoites under the light microscope at 10X and 40X. Then, a few drops of iodine solution were added to samples on glass slides and were covered with a coverslip. The presence of IPIs larvae, ova, and cysts was observed under the light microscope at 10X and 40X magnifications [24].

### 2.10. Formol-Ether Concentration Technique

A portion of each stool sample was used for the detection of parasitic ova and protozoan cysts using the formol-ether concentration technique based on the WHO guideline [24]. One gram (1 g) of each stool sample was first emulsified with 3–4 ml of 10% formalin saline. An additional 3–4 ml of 10% formol saline was added and mixed thoroughly and passed through gauze. Three to four (3–4) ml of diethyl ether was added and mixed by inverting and intermittent shaking of test tubes for 1 minute and centrifuged at 3,000 rpm for 5 minutes. After centrifugation, the layers of fecal debris were detached from the side of the tube using an applicator stick. The fecal debris and the formol saline were discarded and the sediment remained at the bottom of the test tube. The sediment was then transferred to a slide and covered with a coverslip. The preparation was examined microscopically using the 10X and 40X objectives for the identification of cysts and eggs.

### 2.11. Data Analysis

Statistical package for social science (SPSS) version 25.0 (IBM SPSS Statistics) was used to analyze the collected data. Chi-square (*χ*^2^) test was performed to verify the possible association between the prevalence of IPIs and sociodemographic characteristics, socioeconomic characteristics, behavioral factors, personal hygiene, and environmental sanitation factors. Logistic regression was used to measure the degrees of association between the prevalence of IPIs and their associated risk factors. In the modeling process, univariate analysis was first done with a 0.25 level of significance to select the candidate variables for multivariable analysis. The variables, with a *P* value of less than 0.25, were qualified and included in the multivariable analysis [25]. A *P* value below 0.05 was considered significant and 95% CI was used to show the accuracy of data analysis.

### 2.12. Ethical Considerations

Before conducting the investigation, the investigator obtained ethical clearance from the Ethical Committee of Science College, Bahir Dar University (S1_File. Pdf). Written consents were obtained from the parents/guardians of children after explaining the purpose and the procedures of the study. The laboratory test was conducted with strict privacy and confidentiality. Finally, albendazole, mebendazole, and praziquantel drugs were given to the students whose test results were positive by the nurses free of charge.

### 2.13. Data Quality Control

The questionnaire was first pretested using thirty-five individuals outside the study population in other schools to avoid all ambiguities. All ambiguities were corrected before the questionnaires were administered to the actual study participants (S2_File. Pdf). During questionnaire surveys, the principal investigator cross-checked every questionnaire to check whether it was correctly filled by data collectors or not. The effectiveness of the reagents (formalin, diethyl ether, normal saline, and iodine solution) was pretested before starting the diagnosis of the study subjects by the laboratory technicians. A parasitological Atlas was consulted to ensure the accurate identification of the parasite species [26].

## 3. Results

### 3.1. Sociodemographic Characteristics of Study Participants in Dera District 2019/2020

Table 1 depicts the sociodemographic characteristics of the study participants. Of the 384 randomly identified participants, two students (0.5%) were not voluntary to give stool samples and did not fill the structured questionnaire. Hence, 382 (99.5%) (50% male, 50% female) students participated in this study (response rate of 99.5%). The age of the participants ranged from 6 to 21 years. Two hundred fifty-four (66.5%) individuals fall in the age group 12 to 18 years, 123 (32.2%) in 6 to 11 years, and five (1.3%) in 19 to 21 years. Three hundred eight participants (80.63%) were from family sizes of more than five and 74 (19.37%) from less than or equal to five. The water sources of 67.8% of the students were from unprotected sources like springs, wells, and rivers. More than half (50.8%) of the students did not wear shoes.

### 3.2. Prevalence of IPIs

Of the 382 students examined for IPIs, 238 (62.3%; 95% CI: 54.6, 70.7) were positive for one or more IPIs. The gender distribution of IPI positives was 118 (61.8%; 95% CI: 51.14, 73.98) males and 120 (62.8%; 95% CI: 52.09, 75.13) females. The single, double, and triple IPIs were (136, 35.6%; 95% CI: 29.9, 42.11), (98, 25.7%; 95% CI: 20.83, 31.26), and (4, 1.05%; 95% CI: 0.29, 2.68), respectively. Nine parasites were detected using wet-mount and formol-ether concentration techniques (Table 2). *Entamoeba* sp. was the predominant species (113, 29.6%) followed by hookworm (83, 21.7%), *S. mansoni* (43, 11.3%), *Taenia* sp. (35, 9.2%), *G. lamblia* (20, 5.2%), *A. lumbricoides, H. nana*, *E. vermicularis* (16, 4.2% each), and *T. trichiura* (2, 0.5%). The prevalence of protozoan and helminths parasitic infections in the study area was 126 (33%) and 171 (44.76%), respectively.

### 3.3. Univariate and Multivariate Logistic Regression Analysis (LRA) of the Most Important Risk Factors for IPIs

The most important risk factors for IPIs among primary school children in the Dera district were identified using univariate and multivariate logistic regression analyses (MLRA) (Table 3).

Univariate logistic regression analyses showed that statistical differences in IPIs resulted due to variations in school, family size, modes of school waste disposal, knowledge of IPIs, playing with soil, suckling fingers, school toilet function, school water access, and household toilet (*P* < 0.25). The odds of IPIs were higher in Korata Primary School (COR = 3.13; 95% CI: 1.544, 6.344) than others, in students from schools openly disposing of wastes (COR = 2.167; 95% CI: 1.39, 3.375) than burying/burning, among students lack IPI knowledge (COR = 2.245; 95% CI: 1.371, 3.68) than those who knew, and among students who had school water access (COR = 2.112; 95 CI: 1.283, 3.5) than not.

In the multivariate regression, family size more than five, open school waste disposal, and lack of knowledge were more likely associated with IPIs (*P* < 0.05). Students from the family size above five (AOR = 1.8; 95% CI: 1.004, 3.13) were more likely to be infected by IPIs than those from five and below, students from schools where wastes were openly disposed of (AOR = 15.88; 95% CI: 1.91, 132.1) were prone to IPIs compared to those from schools which bury/burn wastes, and students who had no IPI knowledge were subjected to IPI (AOR = 1.8; 95% CI:1.1, 3.2) more than those known.

### 3.4. Risk Factors Associated with *Entamoeba* sp., *G. lamblia*, *E. vermicularis*, *Taenia* sp., and *A. lumbricoides*

*Entamoeba* sp.*, G. lamblia, E. vermicularis, Taenia* sp., and *A. lumbricoides* were the most frequent IPIs detected from the study participants. Their risk factors and association are presented in Table 4.

### 3.5. Risk Factors Associated with *Entamoeba* sp. Infection

The multivariate regression analysis shows that drinking river water, eating soil, and lack of school water access were significantly associated with *Entamoeba* sp. infection (*P* < 0.05) (Table 4). Students who used to drink river water (AOR = 2.35; 95% CI: 1.102, 5.866) were at high risk of *Entamoeba* sp. infection than those who used to drink hand-dug well water, students who ate soil (AOR = 3.96; 95% CI; 1.34, 11.67) were more prone to *Entamoeba* sp. than their counterparts, and students from schools which lack water access (AOR = 1.773; 95% CI: 1.004, 3.13) were more likely infected by *Entamoeba* sp. than students from schools where there is enough water.

### 3.6. Risk Factors Associated with *G. lamblia* Infection

Maternal handwashing habits and school toilet functions were the only predictors of *G. lamblia* in the multivariable analysis (*P* < 0.05) (Table 4). Students from mothers who used to wash their hands with water alone, students from mothers who did not wash their hands, and students who did not use the school toilet were strongly associated with *G. lamblia* (*P* < 0.05). Students from mothers who used to wash their hands with water alone (AOR = 7.973; 95% CI: 0.97, 64.4) and students from mothers who did not wash their hands (AOR = 2.1; 95% CI: 0.17, 25.1) were subjected to *G. lamblia* as compared to children from mothers who used to wash their hands with soap. Students who did not use the school toilet (AOR = 4.13, 95% CI: 1.3, 13.03) were prone to *G. lamblia* more than students who used to.

### 3.7. Risk Factors Associated with *E. vermicularis* Infection

The household drinking water source was the only risk factor associated with *E. vermicularis* (*P* < 0.05) in the multivariable analysis (Table 4). Students who used to drink locally filtered water (AOR = 1.378; 95% CI: 0.188, 10.095) were more likely to be infected with *E. vermicularis* than students who used to drink chlorinated water.

### 3.8. Risk Factors Associated with *Taenia* sp. Infection

Statistically significant differences in *Taenia* sp. (*P* < 0.05) were seen with differences in grade levels and school toilet functions in the multivariate logistic regression analysis (Table 4). Grades 1–4 and not using the school toilet were associated with *Taenia* sp. infection. Students from grades 1–4 (AOR = 2.28; 95% CI: 1.08, 4.814) were more likely to be infected by *Taenia* sp. than students from grades 5–8 and students who did not use school toilet (AOR = 2.651; 95% CI: 1.176, 5.973) were more subjected to *Taenia* sp. than children who did use the school toilet.

### 3.9. Risk Factors Associated with *A. lumbricoides* Infection

Only the habit of eating raw meat resulted in statistically significant differences in *A. lumbricoides* infection (*P* < 0.05) (Table 4). Children who ate raw meat (AOR = 15.282; 95% CI: 1.514, 154.233) were more likely to be infected by *A. lumbricoides* than children who did not eat raw meat.

## 4. Discussion

The overall prevalence of IPIs in the present study was 62.3%. It was comparable with the studies done in Sasiga District, Southwest Ethiopia (62.4%) [27], Sudan (56.9%) [28], Bahir Dar, Ethiopia (65.5%) [29], Southern Ethiopia (56%) [30], and Jawi town, North West Ethiopia (57.88%) [19].

This overall prevalence was higher than that of the national prevalence of IPIs among school children (52%) [31], Senbete and Bete Towns, North Shoa, Ethiopia (52.3%) [32], Homesha District, Northwest Ethiopia (35.44%) [33], Birbir town, Southern Ethiopia (27.1%) [34], Delo-Mena District, Southeastern Ethiopia (26.6%) [35], Adigrat town, Northern Ethiopia (50.81%) [36], Gurage Zone, Ethiopia (40.2%) [37], Bahir Dar, Ethiopia (45%) [38], Gobgob Primary School, Northwest Ethiopia (30.8%) [39], East Arsi, Ethiopia (27.1%) [40], Iran (21.5%) [18], India (27.5%) [41], Western Saudi Arabia (12%) [42], Nigeria (13.7%) [43], Rwanda (44.8%) [44], and Senegal (35%) [45]. But, it was lower than the finding from Mizan-Aman Town, Southwest Ethiopia (76.7%) [46]. These differences could be attributed to the differences in the socioeconomic status of the people, sociodemographic distinctions, knowledge, hygiene, and sanitary facilities, weather, climate, and environmental factors.

The prevalence of IPIs among the male and female students in the Dera District was 61.8% and 62.8%, respectively. No statistically significant difference was observed between male and female students. The outcome of the study was in line with the studies done in Glomekeda district, Northern Ethiopia, males (31.3%) and females (28.6%) [47]; Sasiga District, Southwest Ethiopia, males (62.6%) and females (62.2%) [27]; Bahir Dar, Ethiopia, males (67.8%) and females (63.2%) [29]; Jawi Town, Ethiopia, males (51.85%) and females (45.30%) [19]; and Adigrat town, Northern Ethiopia, males (49.13%) and females (52.94%) [36], *P* > 0.05.

However, it disagrees with the studies done in Gobgob Northwest, Ethiopia, where males (38.9%) were more infected than females (26.4%) *P* < 0.05 [39]. This equal chance of IPIs among males and females may be due to the narrowing of gender role differences in the study area.

Korata, Mirafe Mariam, and Wagira primary schools are found near Lake Tana. Their soil is wet, swampy, and suitable for agriculture and irrigation activities. The people from these areas are farmers and their children are engaged in farming and irrigation activities. Wagira has one dam that is used for irrigation cultivating vegetables, fruits, onions, khat, and cereals. Accordingly, the prevalence of IPIs was high in Korata (74.3%), Mirafe Mariam (61.5%), and Wagira (58.6%).

The prevalence of IPIs in Korata (74.3%) was relatively similar to the studies done in Mizan-Aman town, Ethiopia (72.9%), Ediget Behibret, Ethiopia (88.9%), and Mizan Number One, Ethiopia (83.4%) [46]. The prevalence of IPIs in Mirafe Mariam (61.5%) and Wagira (58.6%) was close to the studies in Cambodia: Dontri locality (54.3%) and Kon Kaêk locality (45.5%) [48]. The result was higher than the studies done in Gurage Zone, Enemorena-Ener District, Ethiopia (40%) [37], Gurage Zone, Abeshege Districts, Ethiopia (38.7%) [37], Mizan-Aman town, Ethiopia (42.9%), Mizan Misgana Acadamy, Ethiopia (35.5%), and Aman Misgana Academy, Ethiopia (30.0%) [46]. However, it was much lower than the study done in Mizan-Aman town, Gacheb School (90%) [46].

The prevalence of protozoa parasitic infections in our study was 33%. The outcome of the study was higher than the studies done in Birbir town, Southern Ethiopia (7.1%) [34], Gobgob, Northwest Ethiopia (7.32%) [39], Sasiga District, Southwest Ethiopia (6.8%) [27], Adele town, East Arsi, Ethiopia (17.7%) [40], Jawi town, Northwest Ethiopia (25.8%) [19], Iran (21%) [18], Sri Lanka (18.4%) [49], and Adigrat town, Northern Ethiopia (6.8%) [36].

On the contrary, the outcome was comparable with the studies conducted in Glomekeda District, Northern Ethiopia (27.8%) [47], and Bahir Dar, Ethiopia (35.9%) [29]. However, it was lower than the studies from Senegal (93.4%) [45] and Sudan (54.2%) [28]. The contradictory reports on the prevalence of protozoan infections could be due to variations in geography, socioeconomic conditions (unprotected source of drinking water, poor personal and environmental sanitation, and accessibility and use toilets), and the methods employed for stool examination, and the time of the study.

The prevalence of helminths in the study area was 44.76%. The result was lower than the studies done in Malaysia (60.8%) [50] and Sasiga District, Southwest Ethiopia (61.1%) [27]. However, it was higher than the studies done in Sudan (2.80%) [28], Iran (0.36%) [18], Senegal (2.2%) [45], Jawi Town, Northwest Ethiopia (32%) [19], Babble town, Eastern Ethiopia (13.8%) [10], Birbir town, Southern Ethiopia (25.4%) [34], Glomekeda district, Northern Ethiopia (12.08%) [47], Adele town, East Arsi, Ethiopia (26.4%) [40], and Gobgob Northwest, Ethiopia (23.44%) [39]. But, it was comparable to Bahir Dar, Ethiopia (50.14%) [29], and Adigrat town, Northern Ethiopia (44.013%) [36]. The variation could be because of differences in socioeconomic distinction, the habit of wearing shoes, participation in agricultural activities, the habit of swimming, and eating uncooked/raw vegetables and foods.

Nine species of IPIs were isolated from the study area. *Entamoeba* sp. (29.6%) was the most predominant parasite followed by hookworm (21.7%), *S. mansoni* (11.3%), *Taenia* sp. (9.2%), *G. lamblia* (5.2%), *A. lumbricoides, H. nana,* and *E. vermicularis* (4.2% each), and *T. trichiura* (0.5%) (Table 2).

The prevalence of *Entamoeba* sp. (29.6%) in the present study was relatively similar to the studies conducted in Bahir Dar, Ethiopia (24.5%) [29], Tanzania (31%) [51], Sudan, (31.2%) [28], and Rwanda (25.95%) [44]. However, it was higher than the study done in Gobgob, Northwest Ethiopia (13%) [39], Sudan (15.50%) [17], Homesha District, Western Ethiopia (14.17%) [33], Jawi town, Ethiopia (5.9%) [19], Cambodia (17.5%) [48], Birbir town, Southern Ethiopia (2.6%) [39], Delo-Mena District, Southeastern Ethiopia (7.7%) [35], Sasiga District, Southwest Ethiopia (8.1%) [27], Adigrat town, Northern Ethiopia (4.5%) [36], Glomekeda district, Northern Ethiopia (19.43%) [47], Adele town, East Arsi, Ethiopia (10.3%) [40], and western Saudi Arabia (2%) [42]. But, it was lower than the study conducted in Nigeria (39.5%) [43]. The difference in the prevalence of *Entamoeba* sp. among the different studies might be due to the differences in the levels of contamination of drinking water sources, consumption of raw vegetables, and handwashing habits of the study participants [52].

The prevalence of *G. lamblia* (5.2%) in the study area was lower than the studies done in Bahir Dar Ethiopia (11.4%) [29], Homesha District, Northwest Ethiopia (12.65%) [33], Jawi town, Ethiopia (19.95%) [19], Gobgob, Northwest Ethiopia (11%) [39], Iran (11.0%) [53], Nigeria (19.7%) [43], Rwanda (19.6%) [44], Sudan (22.9%) [28], Tripoli, Lebanon (28.5%) [54], Cambodia (31.5%) [48], and Senegal (72.5%) [45].

On the other hand, *G. lamblia* prevalence (5.2%) was relatively close to the findings in Delo-Mena (2.0%) [35], Adigrat town, Northern Ethiopia (2.29%) [36], Sasiga District, Southwest Ethiopia (6.5%) [27], Birbir town, Southern Ethiopia (4.8%) [34], Glomekeda district, Northern Ethiopia, (8.29%) [47], and western Saudi Arabia (3%) [42]. But, it was higher than the study from Iran (1.66%) [18]. These variations might be due to variations in the quality of drinking water sources, foods, utilization of toilets, washing hands, and environmental conditions.

The prevalence of hookworm in the study area was 21.7%. This result was relatively similar to the studies done in Bahir Dar, Ethiopia (22.8%) [29], Birbir town, Southern Ethiopia (19.54%) [34], Sasiga District, Southwest Ethiopia (20.6%) [27], and Nigeria (19.7%) [43]. The outcome of this study was higher than in Delo-Mena district (0.8%) [35], Dera Woreda, Ethiopia (14.7%) [55], Homesha District, Northwest Ethiopia (10.12%) [33], Jawi town, Ethiopia (13.8%) [19], Gobgob, Northwest Ethiopia (8.3%) [39], Glomekeda district, Northern Ethiopia (0.7) [47], and Malaysia (4.1%) [50]. But, it was lower than the study done in Nigeria (45.62%) [56], and Bahir Dar, Ethiopia (54.5%) [57]. These reported variations in the prevalence of hookworm infection among studies could be attributed to differences in environmental sanitation, mass treatment, school deworming, and shoes wearing habits of the students.

The prevalence of *S. mansoni* in the study area was 11.3%. The outcome was relatively similar to the outcome of the Delo-Mena district (9.6%) [35], Jawi town, Ethiopia (10.3%) [19], and Bahir Dar Zuria, Ethiopia (15.7%) [57]. However, it was higher than the studies done in Gurage Zone, Ethiopia (1.7%) [37], Homesha District, Northwest Ethiopia (0.75%) [33], Sudan (2.40%) [17], Birbir town, Southern Ethiopia (1.1%) [34], and Sasiga District, Southwest Ethiopia (4.4%) [27]. The possible reason for the high prevalence of *S. mansoni* infection in the present study might be that children have contact with river water during bathing, swimming, fetching water for home use, and irrigation activities. The study area is known for irrigation activities and children participate in agricultural activities to help their parents. The district has rivers (Gumara and Gelda), Lake Tana, Wagira Dam, and other smallest springs used as water sources.

The prevalence of *Taenia* sp. (9.2%) was in line with the studies done in Nigeria (5.3%) [43], Malaysia (9.5%) [50], and Gobgob, Northwest Ethiopia (7%) [39]. However, it was higher than that of Delo-Mena district, Ethiopia (0.2%) [35], Gurage Zone, Ethiopia (1.6%) [37], Homesha District, Northwest Ethiopia (1.77%) [33], and Jawi town, Ethiopia (3%) [19]. Consumption of raw meat and vegetables, drinking water from unprotected water sources, playing with soil, grade level, presence of domestic animals, the habit of sucking fingers, the habit of open defecation, and participating in agriculture activities might be reasons for *Taenia* sp. infection.

Prevalence of *A. lumbricoides* (4.2%) was similar to the studies done in Nigeria (7.9%) [44], Delo-Mena district, Ethiopia (3.7%) [35], Gurage Zone, Ethiopia (9.4%) [37], Birbir town, Southern Ethiopia (8.8%) [34], Bahir Dar Zuria, Ethiopia (8.6%) [57], and Glomekeda district, Northern Ethiopia (3.3%) [47]. The result was lower than the study conducted in Bahir Dar, Ethiopia (13.6%) [29], Nigeria (26.8%) [56], Malaysia (34.3%) [50], Gobgob Northwest Ethiopia (33.3%) [39], Southern Ethiopia (33.2%) [30], Sasiga District, Southwest Ethiopia (22.7%) [27], Adigrat town, Northern Ethiopia (19.1%) [36], Adele town, East Arsi, Ethiopia (12%) [40], and Southwestern China (25.2%) [58]. But, it was higher than studies from Homesha District, Northwest Ethiopia (0.5%) [33], Jawi town, Ethiopia (0.73%) [19], and Sudan (1.20%) [17]. The possible reason for these differences might be explained due to differences in eating raw vegetables, playing with soil, not washing hands before food, and after latrine, participation in agricultural activities, knowledge of IPIs, school waste disposal habit, drinking waters from unprotected sources, and environmental sanitation problems.

*Hymenolepis nana* prevalence in the study area was 4.2%. The result was relatively similar to the studies from Sudan (2.8%) [28], Bahir Dar, Ethiopia (4.7%) [29], Delo-Mena District Ethiopia (5.3%) [35], Homesha District, Northwest Ethiopia (1.26%) [33], Jawi town, Ethiopia (4.2%) [19], Birbir town, Southern Ethiopia (1.7%) [34], and Sasiga District, Southwest Ethiopia (5.7%) [27]. However, it was lower than from Sudan (19%) [17] and Gobgob, Northwest Ethiopia (14%) [39]. The outcome was higher than the study from the Glomekeda district, Northern Ethiopia (0.7%) [47]. These differences might be due to differences in animal contact especially with cats and dogs, eating raw vegetables, personal hygiene, the habit of playing with soil, the habit of sucking fingers, and eating raw meat.

The prevalence of *E. vermicularis* in the study area was 4.2%. The outcome was similar to the studies from Delo-Mena district, Ethiopia (1.2%) [35], Gobgob, Northwest Ethiopia (7%) [39], Sudan (1.20%) [17], Homesha district, Northwest Ethiopia (1.77%) [33], and Adigrat town, Northern Ethiopia (3.56%) [36]. However, it was higher than the prevalence from Iran (0.07%) [18], Birbir town, Southern Ethiopia (0.6%) [34], and Bahir Dar, Ethiopia (0.3%) [29]. The outcome was lower than the study done in China (54.86%) [59]. Drinking water from unprotected sources, sucking fingers, and learning materials, and eating raw vegetables might be reasons for *E. vermicularis* infection in the study area.

The prevalence of *T. trichiura* in the study area was 0.5% and it was the lowest among the nine species identified from the study area. The study was relatively similar to the studies done in Nigeria (3.57%) [60], Delo-Mena district, Ethiopia (1.6%) [35], Gobgob, Northwest Ethiopia (4.8%) [39], Homesha district, Northwest Ethiopia (0.75%) [33], Birbir town, Southern Ethiopia (5.7%) [34], Adigrat town, Northern Ethiopia (3.24%) [36], Bahir Dar, Ethiopia (2.8%) [29], Gurage zone, Southcentral Ethiopia (5%) [37], Bahir Dar Zuria (3%) [57], and Glomekeda district, Northern Ethiopia (0.5%) [47]. However, it was lower than other studies reported from Sasiga District, Southwest Ethiopia (7.6%) [27], Nigeria (14.4%) [56], Malaysia (53.8%) [50], Southwestern China (25.2%) [58], Gedeo Zone, Southern region of Ethiopia (75.2%) [30], and East Arsi, Ethiopia (8.4%) [40]. The variation in *T. trichiura* infection could be attributed to variations in feces disposal (use feces as fertilizer in agriculture), working on a farm, and contamination of food and water.

The predominant IPIs in the five primary schools were *Entamoeba* sp., hookworm, and *S. mansoni*. The prevalence of hookworm was 33%, 22%, 31%, 14.7%, and 17.1% among students from Gibtsawit, Enbosa Maseria, Wagira, Mirafe Mariam, and Korata primary schools, respectively. Likewise, the respective prevalence of *Entamoeba* sp. among Gibtsawit, Enbosa Maseria, Wagira, Mirafe Mariam, and Korata primary school students was 30%, 30%, 25.9%, 25.7%, and 35.2%. *S. mansoni* prevalence was 16.7%, 15.5%, 13.4%, 6.4%, and 6% among students from Gibtsawit, Wagira, Korata, Mirafe Mariam, and Enbosa Maseria primary schools, respectively. These differences might be due to differences in walking barefoot, eating raw vegetables, drinking and the use of water from unprotected sources, swimming in contaminated water bodies, participation in agricultural and irrigation activities, plying with soil, using feces as fertilizers, open field defecations, and handwashing habits.

The present study showed that family size was strongly associated with IPIs. The likelihood of being infected by IPIs among students belonging to a family size of above five was twofold compared to students belonging to a family size of less than or equal to five. The present finding agrees with other studies conducted in Ethiopia [29, 37, 38]. Increasing the family size may increase overcrowding that in turn may lead to contamination with each other. Besides, families with large sizes may face economic problems so that their members may face undernutrition, poor sanitation, poor medication, and poor personal hygiene.

Students who lack knowledge of IPIs were twofold more likely to be infected than students who knew IPIs. This finding agrees with the studies done in Malaysia [50, 61]. The possible reason might be that students who did not know may have practiced activities that expose them to IPIs. Similarly, students may not protect their personal and environmental hygiene, could play with the soil, and practice open field defecation.

Open field school waste disposal activities in the study area were strongly associated with the prevalence of IPIs. Students from schools that practiced open field waste disposal activities were sixteen times prone to IPIs compared to students from schools that did not openly dispose of wastes. The outcome agrees with the study from Ethiopia [33]. Open field waste disposal activity might be a source of infection since students may touch wastes while playing on the ground. In the study area, 28.8% of the schools practiced open field waste disposal activities.

The odds of having *Entamoeba* sp. infection among school children who used to drink river water were twofold higher than that of their counterparts. This was in line with the studies reported by Hailegebriel and Nasiri et al. [29, 52]. River waters might be contaminated by flooding and wastes from domestic animals. Likewise, students who ate soil were fourfold more likely to be infected by *Entamoeba* sp. than their counterparts. This might be because the soil could contain amoeba cysts. However, geophagia was not previously reported as a risk factor for *Entamoeba* sp. infection.

Mothers' handwashing by water alone and not using school toilets by students were risk factors associated with *G. lamblia* infection. The odds of having *G. lamblia* infection among students whose mothers did wash their hands with water alone were eightfold higher than students whose mothers used to wash their hands with soap and water. On the other hand, students from mothers who did not wash their hands at all were twofold more likely to be infected with *G. lamblia* than their counterparts. This might be due to the fact unwashed hands could have a cyst of *G. lamblia*. Washing hands only by water may not clean the microcyst of *G. lamblia* from their hands.

Students who did not use the school toilet were four times more likely to be infected by *G. lamblia* than students who used the school toilet. Open defecation in the school compound might result in infection by *G. lamblia* while students play in the school compound, or perform outreach activities.

Students who used to drink unprotected water were 1.378 times more likely to be infected by *E. vermicularis* than students who used to drink chlorinated and boiled water (protected water). Drinking water sources might be infected due to open field defecation, human and animal contact, and wind. The outcome disagreed with the studies reported in Thailand, China, and Marshall Islands [59, 62, 63].

Grade and the school toilet function were risk factors for *Taenia* sp. (Table 4). The odds of having *Taenia* sp. in grades 1–4 was twofold more likely to be infected than that of students in grades 5–8. Being free to play on the ground, eating unwashed vegetables and fruits, eating raw meat, drinking raw milk, and poor personal hygiene among children who were in grades 1–4 may be the possible reasons for their infection.

Not using the school toilet was associated with a *Taenia* sp. infection. *Taenia* sp. infection increased threefold in students who did not use school toilets compared to students who used school toilets. Children who used open defecation in their school compounds may have disseminated *Taenia* sp. infection to their healthy friends. All schools in the study area did not have handwashing facilities after toilet use. The ova and proglottids of *Taenia* sp. might pass from student to student during playing.

Eating raw meat was the major associated risk factor for *A. lumbricoides* infection in the study area. Children who ate raw meat were fifteen times more likely to be infected by *A. lumbricoides* than children who did not eat raw meat. The study outcome disagrees with the findings from the Gurage zone, Southcentral Ethiopia [37]. Personal hygiene of slaughters and contaminated butchering areas might be the reason for the difference. In Ethiopia, especially in the countryside, there are no specified abattoirs. People perform slaughters on open grounds so that the chance of contamination with soil and soil-transmitted infections may be high.

### 4.1. Limitations

The study was limited to only wet-mount and formol-ether concentration techniques. Not using Kato-Katz, PCR, and Ziehl-Neelsen techniques may underestimate the prevalence of IPIs in the area.

## 5. Conclusions

The prevalence of IPIs is high in the study locality. No clear difference in the prevalence of IPIs exists across the five schools. Family size greater than five, open field school waste disposal activities, and lack of knowledge of IPIs among children are independently associated with the overall prevalence of IPIs. We recommend community-based health education, mass treatment, and school-based deworming without interruption.

## Figures and Tables

**Table 1:** Sociodemographic characteristics of students (*n* = 382) from five primary schools (Gibtsawit Primary school, Enbosa Maseria, Wagira, Mirafe Mariam, and Korata Primary school) in Dera district, Northwest Ethiopia (2020).

Sociodemographic variable	Categories	Frequency	Percent
*Schools*	Gibtsawit Primary School	60	15.7
	Enbosa Maseria	50	13.1
	Wagira	58	15.2
	Mirafe Mariam	109	28.5
	Korata Primary School	105	27.5

*Gender*	Male	191	50
	Female	191	50

*Age (year)*	6–11	123	32.2
	12–18	254	66.5
	19–21	5	1.3

*Grade level*	1–4	162	42.4
	5–8	220	57.6

*Fathers' occupation*	Farmer	379	99.2
	Merchant	0	0
	Government employee	3	0.8

*Mothers' occupation*	Farmer	311	81.4
	Merchant	1	0.3
	Government employee	3	0.8
	Housewife	67	17.5

*Maternal education*	Illiterate	320	83.8
	Primary school	14	3.7
	High school	2	0.5
	Above high school	3	0.8
	Adult education	43	11.3

*Paternal education*	Illiterate	197	51.6
	Primary school	32	8.4
	High school	2	0.5
	Above high school	5	1.3
	Adult education	146	38.2

*Toilet access*	No	230	60.2
	Yes	152	39.8

*Household source of drinking water*	Spring	52	13.6
	River	30	7.9
	Well	177	46.3
	Hand-dug well	123	32.2

*Personal hygiene*	Clean	133	34.8
	Not clean	249	65.2

*Knowledge of IPI*	Yes	295	77.2
	No	87	22.8

*Family size*	≤5	69	18.1
	>5	313	81.9

*The habit of handwashing before a meal*	No	14	3.7
	Yes	368	96.3

*The habit of shoe-wearing*	No	194	50.8
	Yes	188	49.2

**Table 2:** The distribution of intestinal parasite infestation among school students (*N* = 382) in Dera district, Northwest Ethiopia, 2019/2020.

Parasite	Frequency *N* = 382	Gender		Age group			School					Grade	
		Female (*n* = 191)	Male (*n* = 191)	6–11 (*n* = 123)	12–18 (*n* = 254)	19–21 (*n* = 5)	Gib. (*n* = 60)	Enb. (*n* = 50)	Wag. (*n* = 58)	Mir. (*n* = 109)	Kor. (*n* = 105)	1–4 (*n* = 162)	5–8 (*n* = 220)
	+# (%)	+# (%)	+# (%)	+# (%)	+# (%)	+# (%)	+# (%)	+# (%)	+# (%)	+# (%)	+# (%)	+# (%)	+# (%)
Protozoa	126 (33)	65 (34.03)	61 (31.93)	45 (36.6)	81 (31.9)	—	18 (30)	15 (30)	15 (25.9)	36 (33.0)	42 (40)	57 (35.2)	69 (31.4)
*Entamoeba* sp.	113 (29.6)	59 (30.9)	54 (28.3)	41 (33.3)	72 (28.3)	—	18 (30)	15 (30)	15 (25.9)	28 (25.7)	37 (35.2)	52 (32.1)	61 (27.7)
*G. lamblia*	20 (5.2)	8 (4.2)	12 (6.3)	6 (4.9)	14 (5.5)	—	0	0	0	11 (10.1)	9 (8.6)	7 (4.3)	13 (5.9)
Helminths	171 (44.8)	89 (46.6)	82 (42.9)	57 (46.3)	112 (44.1)	2 (40)	28 (46.7)	17 (34)	26 (44.8)	49 (45)	51 (48.6)	80 (49.4)	91 (41.4)
*S. mansoni*	43 (11.3)	19 (9.9)	24 (12.6)	17 (13.8)	26 (10.2)	—	10 (16.7)	3 (6)	9 (15.5)	7 (6.4)	14 (13.4)	22 (13.6)	21 (9.5)
*E. vermicularis*	16 (4.2)	9 (4.7)	7 (3.7)	7 (5.7)	9 (3.5)	—	5 (8.3)	0	3 (5.2)	5 (4.6)	3 (2.9)	9 (5.6)	7 (3.2)
*Taenia* sp.	35 (9.2)	24 (12.6)	11 (5.8)	13 (10.6)	22 (8.7)	—	0	0	0	21 (19.3)	14 (13.3)	20 (12.34)	15 (6.8)
*Hookworm*	83 (21.7)	42 (22)	41 (21.5)	28 (22.8)	54 (21.3)	1 (20)	20 (33.3)	11 (22)	18 (31)	16 (14.7)	18 (17.1)	40 (24.7)	43 (19.5)
*A. lumbricoides*	16 (4.2)	9 (4.7)	7 (3.7)	3 (2.4)	12 (4.7)	1 (20)	1 (1.7)	0	0	9 (8.3)	6 (5.7)	6 (3.7)	10 (4.5)
*T. trichiura*	2 (0.5)	1 (0.5)	1 (0.5)	—	2 (0.8)	—	0	0	0	0	2 (1.9)	0 (0)	2 (0.91)
*H. nana*	16 (4.2)	6 (3.1)	10 (5.2)	2 (1.6)	14 (5.5)	—	1 (1.7)	5 (10)	0	6 (5.5)	4 (3.8)	5 (3.1)	11 (5)
Total (any parasite)	238 (62.3)	120 (62.8)	118 (61.8)	78 (63.4)	158 (62.2)	2 (40)	35 (58.3)	24 (48)	34 (58.6)	67 (61.5)	78 (74.3)	106 (65.4)	132 (60)

Gib. = Gibtsawit Primary School, Enb. = Enbosa Maseria Primary School, Wag. = Wagira Primary School, Mir. = Mirafe Mariam Primary School, and Kor. = Korata Primary School, + = positive, # = number, and (%) = percentage in brackets.

**Table 3:** Univariate and multivariate logistic regression analysis of potential risk factors associated with parasitic infection among school children in Dera district, Northwest Ethiopia, 2019/2020.

Risk factors	Categories	IPIs						
		Total no. (%)	Negative no. (%)	Positive no. (%)	COR, 95% CI	*P* value	AOR, 95% CI	*P* value
*School*	Gibtsawit Primary School	60 (15.7)	25 (41.7)	35 (58.3)	1.52 (0.712, 3.23)	0.024^*∗*^	1.35 (0.602, 3.028)	0.11
	Enbosa Maseria Primary School	50 (13.1)	26 (52)	24 (48)	1		1	
	Wagira Primary School	58 (15.2)	24 (41.4)	34 (58.6)	1.535 (0.716, 3.289)		0.112 (0.012, 1.1)	
	Mirafe Mariam Primary School	109 (28.5)	42 (38.5)	67 (61.5)	1.73 (0.9, 3.4)		0.096 (0.01, 0.8)	
	Korata Primary School	105 (27.5)	27 (25.7)	78 (74.3)	3.13 (1.544, 6.344)		—	

*Family size*	≤5	69 (18.1)	34 (49.3)	35 (50.7)	1	0.030^*∗*^	1	0.049^*∗*^
	>5	313 (81.9)	110 (35.1)	203 (64.9)	1.8 (1.1, 3.03)		1.8 (1.004, 3.13)	

*Mode of school waste disposal*	Burry and firing	119 (31.2)	60 (50.4)	59 (49.6)	1	0.001^*∗*^	1	0.011^*∗*^
	Open field	263 (68.8)	84 (31.9)	179 (68.1)	2.167 (1.39, 3.375)		15.88 (1.91, 132.1)	

*Knowledge of IPIs*	Yes	299 (78.3)	100 (33.9)	199 (67.5)	2.245 (1.371, 3.68)	0.001^*∗*^	1.8 (1.1, 3.2)	0.027^*∗*^
	No	83 (21.7)	44 (50.6)	39 (44.8)	1		1	

*Playing with soil*	No	208 (54.5)	84 (40.4)	124 (59.6)	1	0.24^*∗*^	1	0.148
	Yes	174 (45.5)	60 (34.5)	114 (65.5)	1.287 (0.85, 1.95)		1.5 (0.87, 2.45)	

*Suckling's fingers*	No	130 (34.0)	55 (42.3)	75 (57.7)	1	0.18^*∗*^	1	0.963
	Yes	252 (66.0)	89 (35.3)	163 (64.7)	1.343 (0.871, 2.07)		1.01 (0.59, 1.7)	

*School toilet function*	No	188 (49.2)	57 (30.3)	131 (69.7)	1.869 (1.23, 2.845)	0.004^*∗*^	1.4 (0.825, 2.225)	0.230
	Yes	194 (50.8)	87 (44.8)	107 (55.2)	1		1	

*School water access*	No	105 (27.5)	27 (25.7)	78 (74.3)	2.112 (1.283, 3.5)	0.003^*∗*^	5.3 (0.55, 50)	0.146
	Yes	277 (72.5)	117 (42.2)	160 (57.8)	1		1	

*Household toilet*	No	230 (60.2)	81 (35.2)	149 (64.8)	1.302 (0.9, 1.9)	0.219^*∗*^	1.4 (0.9, 2.204)	0.178
	Yes	152 (39.8)	63 (41.4)	89 (58.6)	1		1	

^*∗*^Statistically significant at *P* < 0.05; 1 = reference value; AOR = adjusted odds ratio; COR = crude odds ratio.

**Table 4:** Univariate and multivariate logistic regression analysis of potential risk factors associated with *Entamoeba* sp., *G. lamblia, E. vermicularis, Taenia* sp., and *A. lumbricoides* infection among school children in Dera district, Northwest Ethiopia.

*Entamoeba* sp.								
Risk factors	Categories	Total no. (%)	Negative no. (%)	Positive no. (%)	COR, 95% CI	*P* value	AOR, 95% CI	*P* value
*Household source of drinking water*	River	30 (7.9)	15 (50)	15 (50)	2.417 (1.07, 5.456)	0.096^*∗*^	2.351 (1.031, 5.360)	0.039^*∗*^
	Spring	52 (13.6)	38 (73.1)	14 (26.9)	0.89 (0.431, 1.839)		0.835 (0.395, 1.764)	
	Well	177 (46.3)	129 (72.9)	48 (27.1)	0.899 (0.54, 1.498)		0.706 (0.395, 1.260)	
	Hand-dug well	123 (32.2)	87 (70.7)	36 (29.3)	1		1	

*Eating soil*	No	367 (96.1)	263 (71.66)	104 (28.3)	1	0.013^*∗*^	1	0.013^*∗*^
	Yes	15 (3.9)	6 (40)	9 (60)	3.79 (1.317, 10.92)		3.957 (1.34, 11.67)	

*School water access*	No	105 (27.5)	68 (64.8)	37 (35.2)	1.439 (0.89, 2.325)	0.137^*∗*^	1.773 (1.004, 3.1)	0.048^*∗*^
	Yes	277 (72.5)	201 (72.6)	76 (27.4)	1		1	

*G. lamblia*								
*Maternal handwashing*	No	85 (22.3)	83 (97.6)	2 (2.4)	1.687 (0.15, 18.99)	0.083^*∗*^	2.09 (.174, 25.1)	0.042^*∗*^
	Yes only by water	226 (59.2)	209 (92.5)	17 (7.5)	5.694 (0.74, 43.56)		7.973 (0.99, 64.5)	
	Yes by soap	71 (18.6)	70 (99)	1 (1)	1		1	

*Taking an antiparasitic drug before 6 months*	No	323 (84.6)	304 (94.1)	19 (5.9)	3.625 (0.476, 27.6)	0.214^*∗*^	3.14 (.39, 25.21)	0.282
	Yes	59 (15.4)	58 (98.3)	1 (1.7)	1		1	

*Knowledge of IPIs*	No	299 (78.3)	281 (94)	18 (6)	2.594 (0.59, 11.42)	0.207^*∗*^	1.735 (.37, 8.08)	0.483
	Yes	83 (21.7)	81 (97.6)	2 (2.4)	1		1	

*School water access*	No	105 (27.5)	96 (91.4)	9 (8.6)	2.267 (0.911, 5.64)	0.078^*∗*^	2.101 (0.78, 5.59)	0.137
	Yes	277 (72.5)	266 (96)	11 (4)	1		1	

*School toilet function*	No	188 (49.2)	172 (91.5)	16 (8.5)	4.419 (1.45, 13.5)	0.009^*∗*^	4.132 (1.3, 13.03)	0.015^*∗*^
	Yes	194 (50.8)	190 (97.9)	4 (2.1)	1		1	

*E. vermicularis*								
*Maternal hand wash*	No	85 (22.3)	84 (98.8)	1 (1.2)	0.411 (0.036, 4.63)	0.212^*∗*^	0.432 (0.034, 5.6)	0.118
	Yes only by water	226 (59.2)	213 (94.2)	13 (5.8)	2.106 (0.464, 9.56)		2.858 (0.53, 15.5)	
	Yes by soap	71 (18.6)	69 (97.2)	2 (2.8)	1		1	

*Household drinking water*	Boiling	3 (0.8)	3 (100)	0 (0)	0.000 (0.000)	0.125^*∗*^	----------	0.030^*∗*^
	Filtering	36 (9.4)	32 (88.9)	4 (11.1)	1.250 (0.209, 7.46)		1.378 (0.19, 10.1)	
	Direct	321 (84.03)	311 (96.9)	10 (3.4)	0.322 (0.07, 1.567)		0.206 (0.038, 1.13)	
	Chlorine treated	22 (5.8)	20 (90.9)	2 (9.1)	1		1	

*Knowledge of IPIs*	No	299 (78.3)	284 (94.9)	15 (5.02)	4.331 (0.56, 33.28)	0.159^*∗*^	5.473 (0.67, 44.69)	0.113
	Yes	83 (21.7)	82 (99)	1 (1)	1		1	

*A habit of eating raw meat*	No	147 (38.5)	144 (97.9)	3 (2.1)	1	0.112^*∗*^	1	0.062
	Yes	235 (61.5)	222 (94.5)	13 (5.5)	2.811 (0.79, 10.04)		3.581 (0.94, 13.7)	

*Taenia* sp.								
*Household source of drinking water*	River	30 (7.9)	28 (93.3)	2 (6.7)	1.686 (0.311, 9.143)	0.106^*∗*^	2.004 (0.353, 11.392)	0.168
	Spring	52 (13.6)	46 (88.5)	6 (11.5)	3.078 (0.89, 10.58)		2.826 (0.793, 10.068)	
	Well	177 (46.3)	155 (87.6)	22 (12.4)	3.350 (1.23, 9.106)		3.441 (1.133, 10.447)	
	Hand-dug well	123 (32.2)	118 (95.9)	5 (4.1)	1		1	

*Gender*	Female	191 (50)	167 (87.4)	24 (12.6)	2.352 (1.117, 4.95)	0.024^*∗*^	2.098 (.971, 4.535)	0.059
	Male	191 (50)	180 (94.2)	11 (5.8)	1		1	

*Grade*	1–4	162 (42.4)	142 (87.7)	20 (12.3)	1.925 (0.95, 3.89)	0.068^*∗*^	2.28 (1.08, 4.814)	0.031^*∗*^
	5–8	220 (57.6)	205 (93.2)	15 (6.8)	1		1	

*A habit of sucking fingers*	No	130 (40)	122 (93.8)	8 (6.2)	1	0.148^*∗*^	1	0.408
	Yes	252 (60)	225 (89.3)	27 (10.7)	1.83 (0.807, 4.152)		1.44 (0.607, 3.417)	

*School water access*	No	105 (27.5)	91 (86.7)	14 (13.3)	1.875 (0.915, 3.84)	0.086^*∗*^	1.021 (0.415, 2.514)	0.964
	Yes	277 (72.5)	256 (92.4)	21 (7.6)	1		1	

*School toilet function*	No	188 (49.2)	163 (86.7)	25 (13.3)	2.822 (1.316, 6.053)	0.008^*∗*^	2.651 (1.176, 5.973)	0.019^*∗*^
	Yes	194 (50.8)	184 (94.8)	10 (5.2)	1		1	

*A. lumbricoides*								
*Age*	6–11	123 (32.2)	120 (97.5)	3 (2.4)	0.1 (0.008, 1.185)	0.180^*∗*^	0.012 (0.00, 2.25)	0.251
	12–18	254 (66.5)	242 (95.3)	12 (4.7)	0.198 (0.02, 1.91)		0.017 (0.0, 2.856)	
	19–21	5 (1.3)	4 (80)	1 (20)	1		1	

*Household source of drinking water*	River	30 (7.9)	27 (90)	3 (10)	13.556 (1.4, 135.4)	0.099^*∗*^	13.837 (1.1, 174.7)	0.158
	Spring	52 (13.6)	51 (98.1)	1 (1.9)	2.392 (0.15, 38.98)		2.542 (0.128, 50.6)	
	Well	177 (46.3)	166 (93.8)	11 (6.2)	8.084 (1.03, 63.46)		7.677 (0.78, 75.5)	
	Hand-dug well	123 (32.2)	122 (99.1)	1 (0.9)	1		1	

*A habit of eating raw meat*	No	147 (38.5)	146 (99.3)	1 (0.7)	1	0.027^*∗*^	1	0.021^*∗*^
	Yes	235 (61.5)	220 (93.6)	15 (6.4)	9.955 (1.3, 76.177)		15.282 (1.5, 154.2)	

*Knowledge of IPIs*	No	299 (78.3)	284 (94.9)	15 (5.1)	4.331 (0.56, 33.3)	0.159^*∗*^	17.284 (0.4, 721.8)	0.134
	Yes	83 (21.7)	82 (98.8)	1 (1.2)	1		1	

*Mode of school waste disposal*	Open field	263 (68.8)	248 (94.3)	15 (5.7)	7.137 (0.932, 54.674)	0.059^*∗*^	4.2 (0.51, 34.94)	0.184
	Burry and firing	119 (52.1)	118 (99.2)	1 (0.8)	1		1	

^*∗*^Statistically significant at *P* < 0.05; 1 = reference value; AOR = adjusted odds ratio; COR = crude odds ratio.

## Data Availability

The data supporting the conclusions of this article are within the article and the supplementary file.

## Supplementary Materials

Supplementary Materials S1_File. Pdf. Ethical clearance paper. S2_File. Pdf. Questionnaire (English and Amharic versions).
