# Supplementary material for: Prevalence and Risk Factors Associated with Intestinal Parasitic Infection among Primary School Children in Dera District, Northwest Ethiopia
Source: Can J Infect Dis Med Microbiol. 2021 Sep 21;2021:5517564. doi: 10.1155/2021/5517564 (PMC8478561; doi:10.1155/2021/5517564)
Supplement: Supplementary Materials — S1_File. Pdf. Ethical clearance paper. S2_File. Pdf. Questionnaire (English and Amharic versions). [file 5517564.f1.zip › 5517564.f1/S1_File. Pdf.pdf]

ሳይንስ ኮሌጅ
የድህረ ምረቃ፣ምርምርና ማህበረሰብ
አገልግሎት ም/ዲን
ባሕር ዳር ዩኒቨርሲቲ
ባሕር ዳር - ኢትዮጵያ

Wisdom at the source of the Blue Nile

Science College
The Graduate, Research
& Community Services V/Dean
Bahir Dar University
Bahir Dar – Ethiopia

✉ 79
251 (582) 226 6597
ፋክስ Fax: 251 (582) 220- 20- 25
e-mail: negatassie@yahoo.com
website: www.bdu.edu.et

ቁጥር: PGRCSV[illegible]/[illegible]/2012
ቀን : 08/07/2012 ዓ.ም

Ethical Clearance Approval Form

Applicant's Name: Dires Tegen

Research Title	Prevalence and associated risk factors of major intestinal parasitic infections among primary school children in Dera District, northwest Ethiopia
Researcher (s) Name (s)	Dires Tegen

Thank you for submitting your application for ethical clearance, which was considered at the College of Science Research Ethics Committee meeting on 14 March 2020. The committee has reviewed your ethical application, issues pertaining to participants, consent form, debriefing, and relevant questionnaires.

The researcher should keep the confidentiality of the identity of research participants and data that will be obtained from them. Any serious adverse events or significant changes which occur in connection with this study and /or which may alter its ethical consideration must be reported immediately to the committee for a possible ethical amendment.

We are therefore pleased to inform you that the College's Ethical Clearance Committee has approved your study from an ethical point of view.

With kind regards

CC//

- Dean office
- The Graduate, Research and Community Services V/Dean
- Biology Department

College of Science
