# Supplementary material for: Prevalence and Risk Factors Associated with Intestinal Parasitic Infection among Primary School Children in Dera District, Northwest Ethiopia
Source: Can J Infect Dis Med Microbiol. 2021 Sep 21;2021:5517564. doi: 10.1155/2021/5517564 (PMC8478561; doi:10.1155/2021/5517564)
Supplement: Supplementary Materials — S1_File. Pdf. Ethical clearance paper. S2_File. Pdf. Questionnaire (English and Amharic versions). [file 5517564.f1.zip › 5517564.f1/S2_File. Pdf.pdf]

QUESTIONNAIRES

1.1. English version

Questionnaire for collection of information on the; Prevalence of Intestinal Parasitic Infections and Associated Risk Factors among Schoolchildren in Dera District, North West Ethiopia.

Code no.__________

Part I. Participant identification

Area of residence Woreda ________ Schools________ date______________ Religion--

Part II. Student's profile

1. Sex___ M____ F____ 2. Age_________3. Grade_________4. No. of family------------
5. Fathers occupation; Farmer_____, Merchant_________ Government employee---------
6. Mothers occupation; Farmer_____, Merchant_________ Government employee---------House wife_________
7. Mothers education status: illiterate----, primary school-----, high school----, above high school--- Adult education _________
8. Fathers education status: illiterate----, primary school-----, high school----, above high school--- Adult education _________

Part III. Information on Risk Factors

1. Do you wash your hands always before the meal? How? Yes, only by water ----.Yes, by soap ----No ----
2. Do you wash your hands always after latrine use? How? Yes, only by water ----.Yes, by soap ----No ----
3. Does your Mother wash her hands always before the meal, after latrine use and dispose of baby's feces? How? Yes, only by water ----.Yes, by soap ----No----
4. Do you wear shoes always? Yes------ No------
5. Do you have a household toilet? Yes------ No-----
6. If your answer is yes for question 5 have you use it? Yes ------No------
7. If your answer is yes for question 5 what types of latrine? Private---, Public-----

8. If your answer is no for question 5 where do you defecate? Open field-----near to the river---
9. Where do you get drinking water (source of drinking water) ? Spring----, River-----, Well, ------Hand- dug well------
10. How do you use drinking water? By boiling----, Filtering-----Direct-----, Chlorine treated--
11. How do you dispose of household wastes? Burry underground/Incinerate-----, open Field—, into the river---------Firing--------
12. Are there any domestic animals in your house (Like; Cattle, Sheep, Goat, Dog, Cat, Donkey, Horse, and Mule)? Yes---------No---------
13. Have you ever eaten raw meat? Yes------, No------------
14. What about uncooked vegetables? Yes------, No------------
15. Do you participate in agriculture activities? Yes---------No---------
16. What about your personal hygiene (hygiene and life skills practice)? Clean-----, not clean---------
17. Do you swim or wash your body in the River, Lake, and Pond? Yes---------No---------
18. Do you have the knowledge about IPIs? Yes-------- No---------
19. Do you take any anti-parasitic drug before 6 months? Yes--------, No-----------
20. Is there any deworming program in your school from 2011 to 2012? Yes---------No-----
21. Do you have the habit of playing with soil? Yes---------- No----------
22. Do you have the habit of eating soil? Yes---------- No----------
23. Do you have the habit of sucking your finger, pen, pencils, etc? Yes---------- NO----------

Part IV Schools Information

1. Does the school have a toilet? Yes------- No-------
2. Do you use it? Yes------No------
3. If your answer is No for question 2 where do you defecate? Open field in the school compound----- Open field out of the school compound-----
4. Does the school have water access? Yes------ No------
5. What is the source of water? River------Well------Pond------Pipe------spring------Hand-dug well------
6. Do you drink the school water? Yes------No------

7. If the school didn't have a water source where do you drink when you are thirsty? From the river found near to the school------ Taking from their house---------- From the spring found near to the school------ From the lake found near to the school------ From the Hand-dug well found near to the school------
8. Does the school water treated? How long it was treated? Yes one year ago------ Yes two years------ Yes above two years-------Yes below six months--------- No------
9. How does the school dispose of wastes? Burry underground/Incinerate and firing/ burning -----, open field—,

1.2. Amharic version (የጽሁፍ መጠይቅ የአማርኛዉ ትርጉም)

ክፍል 1. ለተሳታፊዎች የሚሰጥ መለያ

ቀን፡------------------ት/ቤት፡-------------ወረዳ፡-------------የመለያቁጥር፡----------ሀይማኖት--------

ክፍል 2. የተማሪዉ/ዋ መረጃ

1. ፆታ፡- ወንድ---- ሴት ------- 2. ዕድሜ፡-------- 3 የክፍል ደረጀ -----
4. የቤተሰብ ብዛት-------------5. የአባት ስራ፤ ግብርና-----------የመንግስት ሰራተኛ------------ ነጋዴ-------------
6. የእናት ስራ፤ ግብርና------------ነጋዴ------------ የመንግስት ሰራተኛ------------
የቤት እመቤት-----------
7. የእናት የትምህርት ደረጃ፤ ማንበብ መጻፍ የማትችል -----------ጎልማሳ----------የመጀመሪያ ደረጃ----------ሁለተኛ ደረጃ----------ከሁለተኛ ደረጃ በላይ-----------
8. የአባት የትምህርት ደረጃ፤ ማንበብ መጻፍ የማይችል -----------ጎልማሳ----------የመጀመሪያ ደረጃ----------ሁለተኛ ደረጃ----------ከሁለተኛ ደረጃ በላይ-----------

ክፍል 3. የበሽታ መተላለፊያ መንስኤዎች መረጃ

1. ምግብ ከመመገብዎት በፊት እጅዎትን ይታጠባሉ? እንዴት ነዉ የሚታጠቡት? አዎ በዉሀ ብቻ --------አዎ በሳሙና--------የለም----------
2. ሽንት ቤት ከተጠቀሙ በኃላ ሁልጊዜ እጅዎትን ይታጠባሉ? እንዴት ነዉ የሚታጠቡት? አዎ በዉሀ ብቻ --------አዎ በሳሙና--------የለም----------

3. እናትሽ/ እናትህ ምግብ ከማዘጋጀቷ በፊት፣ከሽንትቤት ከተጠቀመች በኂላ እና ህጻናትን ከአጸዳዳች በኂላ ሁልጊዜም እጇን ትታጠባለች? እንዴት ነዉ የሚታጠቡት? አዎ በዉሀ ብቻ --------አዎ በሳሙና--------የለም----------
4. ሁልጊዜ ጫማ ያደርጋሉ? አዎ-------፣ የለም-------
5. ሽንት ቤት አልዎት? አዎ-------፣ የለም-------
6. መልስዎት አዎ ከሆነ ትጠቀሙበታላችሁ? አዎ-------የለም-------
7. ለ5ኛዉ ጥያቄ መልስዎት አዎ ከሆነ ምን አይነት? የግል-------፣ የጋራ-------
8. ለ5ኛዉ ጥያቄ መልስዎት የለም ከሆነ የሚፀዳዱት የት ነዉ? ሜዳ ላይ /ጉዋሮ ላይ/------- ወንዝ ዳር-------
9. ለመጠጥ የሚሆን ውሃ ከየት ነው የሚያገኙት? ከወንዝ------፣ ከምንጭ---- ፣ከጉድጉዋድ------ከፊኒዳ /Hand-dug well/-------
10. ለመጠጥ የሚገለገሉበትን ውሃ በምን ዘዴ ነው የሚጠቀሙበት?
በማፍላት------፣ በማጥለል----፣ በቀጥታ በመጠቀም------፣ በክሎሪን (በዉሀ አጋር) በማከም-----
11. ከቤት የሚወጣን ጠጣርም ሆነ ፍሳሽ ቆሻሻን በምን መልኩ ነው የሚያስወግዱት? ጉድጓድ ውስጥ በመቅበር--------፣ ሜዳ ላይ በመጣል-------፣ ወንዝ ውስጥ በመጣል----በማቃጠል-------
12. ከቤታችሁ የቤት እንስሳት አሉ (ከብት፣ፍየል፣በግ፣ዉሻ፣ድመት እና የጋማ ከብቶች)? አዎ-------፣ የለም-------
13. ጥሬ ስጋ ይመጋባሉ? አዎ-------፣ የለም-------
14. ጥሬ አትክልት ይመጋባሉ (ቲማቲም፣ካሮት፣ጎመን ወዘተ.)? አዎ-------፣ የለም-------
15. በእርሻ ስራ ላይ ይሳተፋሉ? አዎ ------- የለም-------
16. የግል ንጽህና አጠባበቅዎትና የህይወት ልምድዎት እንዴት ነው? ጥንቁቅ----- ፣ቸልተኛ---
17. ወንዝ ላይ ይዋኛሉ ወይም ወንዝ ላይ ገላዎትን ይታጠባሉ ወንዝ አቋርጠዉ ያዉቃሉ? አዎ----- የለም-----
18. በአንጀትጥገኛ ተህዋስያን አማካኝነት ስለሚከሰቱ በሽታዎች ግንዛቤ አለዎት? አዎ እዉቀቱ አለኝ -------፣ የለም እዉቀቱ የለኝም ---
19. ከስድስት ወራት በፊት የፀረ-ጥገኛ ተህዋስያን መድሃኒት ወስደዉ ያውቃሉ? አዎ-------፣ የለም---

20. ከ2011 እስከ 2012 አሁኑ ሰአት ድረስ ትምህርት ቤታችሁ ላይ የፀረ-ጥገኛ ተህዋስያን መድሃኒት ክትባት ተሰጥቷል? አዎ-------የለም-------
21. አፈር ላይ የመጫዎት ልምድ አለህ/ ሽ? አዎ-------የለም-------
22. አፈር የመብላት ልምድ አለህ/ አለሽ? አዎ-------የለም-------
23. እጅህን/ ሽን፤እስክብሪቶ፤ እና እርሳስ ወዘተ. ወደ አፍህ/ ሽ የማስገባት ልምድ አለህ/ ሽ? አዎ------- የለም-------

<u>ክፍል 4. የት/ቤት መረጃ</u>

1. ት/ቤቱ ሽንትቤት አለዉ? አዎ------- የለም-------
2. መልሳችሁ አዎ ከሆነ የት/ቤቱን ሽንትቤትትጠቀሙበታላችሁ? አዎ-------የለም-------
3. ካልተጠቀማችሁበት የት ነዉ የምትጠቀሙት? ት/ቤት ግቢ ሜዳ ላይ---------ከት/ቤት ግቢ ዉጭ ሜዳላይ--------
4. ትቤቱ የዉሃ አቅርቦት አለዉ? አዎ -------የለም-------
5. ትቤቱ ዉሃ ከየትነዉ የሚያገኘዉ? ከወንዝ-----ከጉድጓድ-------ከኩሬ-------ከቧንቧ ዉሃ-------ከምንጭ-------ከፊንዳ/ ሀንዳግ ዌል/-------
6. የትቤቱን ዉሃ ተማሪዎች ትጠቀሙበታላችሁ? አዎ------የለም-------
7. የትቤቱን ዉሃ ተማሪዎች የማትጠቀሙት ከሆነ ዉሃ ከየት ነዉ የምትጠጡት? ከት/ቤቱ አካባቢ ከሚገኘዉ ወንዝ--------ከቤት በማምጣት-----ከት/ቤቱ አካባቢ ከሚገኘዉ ምንጭ-------ከት/ቤቱ አካባቢ ከሚገኘዉ ሀይቅ-------ከት/ቤቱ አካባቢ ከሚገኘዉ ፊኒዳ (ሀንዳግ ዌል)-------
8. የት/ቤቱ ዉሃ ታክሞ ያዉቃል? ከታከመ ምንያክል ጊዜ ሆነዉ? አዎ ከ6ወር በታች-------አዎ 1አመት----አዎ 2አመት-------አዎ ከ2አመት በላይ----------የለም-------
9. ት/ቤቱ ደረቅ አና ፍሳሽ ቆሻሻዎችን እንዴት ነዉ የሚያስዎግደዉ? በመቅበር ፤ በማቃጠል እና በማስረግ-------፣ ሜዳ ላይ በመጣል-------፣
